# New York Pathological Society

**Published:** 1859-07

**Authors:** E. Lee Jones


### ART. V.—*New York Pathological Society.*

REGULAR MEETING, Jan. 26, 1859. JOHN C. DALTON, JR., M. D., President.

(Reported for the Journal, by E. LEE JONES, M. D., Secretary.)

### FATTY TUMOR WEIGHING FIVE POUNDS.—HYPERTROPHY OF ADIPOSE-CELLULAR TISSUE, WITHOUT LOBULE OR CYST.

Dr. George F. Shrady presented a specimen of a large fatty tumor, weighing five pounds, removed from a patient in the New York Hospital, by Dr. Wm. H. Van Buren on the 18th of January.

Mary H———, aged eighteen, a native of Canada, was admitted into the N. Y. Hospital, in the service of Dr. Van Buren, on the 12th of Jan., with a large fatty tumor of the back, and deposits of fat in other parts of her body.

The first appearance of anything of the sort she dated as far back as ten years ago, when she noticed a tumor about the size of a hen's egg on the lower part of the back of her neck. This tumor increased in size, very slowly, for about eight years; when, two years ago, it commenced to grow very rapidly, doubling its former size in that time.

About three years ago she first noticed an unnatural fullness under her chin in the suprahyoideal region; this continued to grow until it left a large pendulous and unsightly mass of fat, which occupied nearly the whole of the anterior cervical region at the time of her admission.

Very soon after she first noticed this tumor under the chin, bunches of fat began to make their appearance on each shoulder; these also at the time of her admission were of very considerable size.

About a year ago, the whole of the back began to be the seat of a pretty evenly diffused fatty mass, which extended from just above the buttocks, and was lost in the enormous tumor alluded to. This condition of things still exists, together with the tumors of the shoulders and under the chin.

She was a patient of the house last August; but, being at that time not in a fit condition to warrant the attempt at an operation, she was discharged without anything having been done for her. From that time until her second admission, thinking that the tendency to the deposit of fat might be arrested by abstaining from animal food, she lived for a considerable length of time on vegetable diet. As a seeming consequence of this, instead of diminishing any such tendency, the tumor began to sensibly increase.

On presenting herself a second time for treatment, her condition was deemed favorable for an operation, at least so far as removing the tumor upon the back was concerned. It was accordingly removed by Dr. Van Buren, on the 18th of this month.

The patient being etherized, a longitudinal incision was made from the upper to the lower limit of the tumor; when it was found that, instead of being composed of any distinct lobules and a cyst, it seemed to be the result merely of a hypertrophy of the subcutaneous adipose tissue in the vicinity. As a consequence of this, the tumor had to be dissected out carefully on all sides, causing the operation to last a full hour and more. She seemed to bear up under the operation very well, so far as any direct shock was concerned. The two immense flaps were brought together with sutures and adhesive straps; while a compress of cotton, corresponding in size to the extent of the tumor, was laid over the whole, and confined by a figure-of-eight bandage. She was directed to lie upon her back, her arms being secured in front, in order to favor union by first intention as far as possible. She went on very well the rest of the afternoon, and passed a quiet night by the aid of an anodyne draught.

She continued to do well the next day, well regulated and moderate pressure being kept up over the former seat of the tumor. On the 20th, two days after the operation, the dressings were removed; when it was found that the site of the tumor was occupied by a cyst, which contained about a pint of bloody serum. Over the external surface of the flaps, blebs had formed; and the flaps themselves were found to be in a sloughy condition. The wound was laid open; and it was found that the whole of the internal surface of the sac was in a sloughy condition. The cavity was syringed out with warm water, and yeast dressings applied; at the same time she was directed to lie upon her side.

In consequence of the condition of her wound, she became very much prostrated, her pulse having risen in twenty-four hours, from 130 to 160 per minute. She was freely stimulated with carb. ammon. in 5-gr. doses; an extra allowance of beef tea ordered; and in addition to all this, sulph. quinine was administered in three-grain doses every four or five hours. By this treatment, in the course of twenty-four hours her pulse decreased in frequency to 140. In the mean time, the slough had limited itself to the extent occupied by the tumor.

From that time until the present, her pulse has been decreasing gradually in frequency, and becoming fuller, until now it is 120. The whole of both flaps have separated, the whole bed of the wound is now covered with granulations, and the discharge looks healthy. She seems to be now in a fair way to recover.

The tumor is an interesting one, not only in relation to its size, but its general character; it being nothing more than a hypertrophy of the adipose-cellular tissue, devoid of any cyst, or distinct lobules.

It seems that the sloughing of the flaps was due in a great measure to the lack of vital force in the patient, in consequence of the deposit of fat throughout the body. Possibly, her position in bed for the first forty-eight hours, helped to bring about the result.

The specimen is interesting on account of its size and general character, as a mere hypertrophy of adipose cellular tissue, without lobules or a cyst.

### ACUTE ABCESS OF LUNG.

Dr. Finnell presented a specimen of acute abscess of the lung in a child seven months old; who died after a few days' illness, the only symptoms that presented themselves being, slight cough, with a little fever. It occurred in the practice of Dr. Peugnet. Postmortem examination revealed a large abscess in the middle lobe of the right lung, without any evidences of any tuberculous deposit in either organ. He thought that it was an unusual specimen, on that account.

Dr. L. A. Sayre stated that the specimen, to him, was an unique one; and he thought it had an important bearing upon the subject that was exciting so much attention in the medical world at that time.

FATTY HEART AND KIDNEY.

Dr. Finnell presented next a *fatty heart and kidneys*, removed from the body of a woman who died in a state of intoxication.

HYPERTROPHIED HEART.

Dr. F. presented a third specimen, a *hypertrophied heart*, with a theromatous deposit in the walls of left ventricle, with an absence of any disease in the artery. The man died suddenly. The pericardium was adherent throughout the whole of its extent.

GUNSHOT WOUND OF THE HEART.

The fourth specimen presented by Dr. F., was a *gunshot wound of the heart*, from one of the victims of the Elm-street tragedy. The ball entered the right side of the heart near the apex, coming out at the base and wounding the aorta; it passed through the pericardium again, by the side of the spinal column, through the left lung.

TUBERCULOUS DEPOSIT COUGHED UP.

Dr. Alonzo Clark presented two specimens of tuberculous deposits coughed up by a girl nine or ten years of age. One of the pieces was the size of a Lima bean; the smaller about as large as an ordinary bean. The father of the child, who is a physician, told Dr. C. that she had a cough for two years, and he had satisfied himself that no tubercles existed. Dr. C. had not yet seen the child.

The child, according to account, was somewhat emaciated; and, with the exception of about three months last summer, coughed daily for the whole of the two years. This cough had become so habitual with the child, that the father began to think but little of it. About three weeks ago, she began to cough extraordinarily for a long time, and finally expectorated the larger of the two masses, with a slight trace of blood in the expectoration. After this she seemed to be very comfortable for three or four days; when the coughing was renewed, and the smaller mass discharged.

Dr. Clark stated that the masses were composed of tuberculous matter; with certain small concretions, some of which effervesced with and were dissolved in nitric acid, and others dissolved without effervescence in the same liquid. The first was the amorphous carbonate of lime; the second appeared to be the triple phosphate.

### EXTRAORDINARY DEVELOPMENT OF TUBERCULOUS MATTER IN LYMPHATIC GLANDS.

Dr. Clark next presented a specimen of what he believed to be an *extraordinary development of tuberculous matter in the lymphatic glands* of a young man sixteen years of age, with the following history:

"The young man was born in Georgia, and had come to the North for his education. Before leaving home, he had intermittent fever many times, and had taken calomel and quinine freely.

"He had been looked upon as a very healthy boy, although for the last year he was noticed to be paler than formerly. He was unusually mature in his judgment and character, and well grown.

"He used to say to his uncle, in October last, that he was short-breathed in swimming and running. After this, he grew pale, and a chain of glands began to be enlarged from the ear to and under the clavicle.

"He began to cough two or three months ago; and the cough gradually increased in severity, till I (Dr. C.) saw him, in December.

"On December 26th, I saw him for the first time. He was now suffering from shortness of breath on any exertion; his complexion was pale and waxen, tissues slightly œdematous, lips had but little color, and strength was a good deal enfeebled. The bowels so swollen that he was compelled to enlarge the waistbands of his trowsers with a string. His appearance suggested the probability of albuminuria. On examination of his bowels, there was a tumor of the epigastric region; from the general characters it presented, it was uncertain whether it was due to some morbid state of the liver, or to that of some tissue below it. No fluctuation could be felt; but there was marked tenderness, which rendered it difficult to effect a thorough exploration.

"There was distinct dullness on percussion under the right clavicle; and throughout the right lung there was crepitant râles, most marked at apex. On left side, slight bronchial effusion. Under left clavicle resonance quite clear.

"At my second visit, shortness of breath continued. The tumor of epigastrium had changed its form somewhat. Sweats at night, but no chills. Dyspnœa sometimes extreme; excessive bronchial râles all over the chest, most on right side; no hemoptysis; spleen large and distinct, reaching nearly to crest of ilium."

From the history of the patient, and the examination made, Dr. C. entertained but little doubts that there were tubercles in the right lung, at apex, somewhat advanced; and, as there was a perceptible shade of dullness throughout the right lung posteriorly, that the tubercles were considerably diffused through it. An opinion regarding the nature of the tumor could hardly be expressed; but taking into account the night sweats, which had been irregular it is true, Dr. C. was inclined to the idea of abscess.

The urine was examined for albumen, and for casts; but neither was found. The fluid seemed to be normal. At the third visit, the patient was paler if possible than before. The bronchial râles were extreme on the right side. Dullness very considerable throughout the whole right chest, most marked, however, under the clavicle. Some œdema of the tissues; bowels still swollen; character of tumor not yet developed; tympanitis and some tenderness; dyspnœa so great, to lie on his back for a moment, to permit an examination of his abdomen, was impossible.

He was accustomed to sleep resting on his knees, and placing a pillow between his thighs and abdomen, and leaning forward on another pillow. This was so striking a feature of his case, that he wishes to give it prominence.

From this time till after death, he did not see him; but was informed that his dyspnœa rather increased till the 19th, when a diarrhea set in, of a bilious character, which the next day was purely mucous, and on the 21st, he discharged nothing but pus. Of this, he passed in the course of the day, by estimate, two quarts, in quantities varying from four to six ounces at a time. This ceased on the 22d in the evening. During the 23d, his breath grew shorter; and he died easily and quietly on the morning of the 24th.

This young man's appetite continued to be good almost to the hour of his death.

The glands of the neck rather enlarged during the time he saw him, and after death were found to be continuous with the groups in the anterior and posterior mediastina. Some of these were as large as an English walnut.

*Autopsy.*—The tegumentary tissues were very thin and watery, but hardly œdematous; peritoneal cavity contained about three pints of thin, sero purulent matter, in which floated shreds of water-soaked fibrine. There was no redness or other evidence of peritoneal inflammation. The intestines were everywhere collapsed, contracted, and nearly empty; no tympanitis present.

Each pleuritic cavity contained about a pint of clear serum; but there was no trace of fibrinous exudation. The pericardium contained about three ounces or so of transparent serum. The liver was of normal size, was of a very dark hue, but contained no morbid deposit. The kidneys could not be said to be diseased; though the left was larger than natural, and pale, while the right was rather congested, being of natural size. The heart appeared to be healthy; this was the case also with the vessels throughout the thorax and abdomen.

The disease was an enormous enlargement of the glands behind the stomach, pancreas, duodenum, transverse colon, and upper part of small intestines. The pyloric orifice of the stomach arched forwards to go over this mass; and the pancreas was nearly imbedded in it. The mass was composed of aggregated tumors varying from the size of a bean to that of a small apple, all of a white or grayish white color. The mass extended from between the crura of the diaphragm, from the last dorsal to the third lumbar vertebra; many small tumors being hidden behind the crura. Laterally it nearly touched the kidneys at the top on each side, and was from three to four inches in thickness on the spine. The mass pressed upward against the inferior surface of the liver; and one large tumor was adherent to this organ, or rather to the gall-bladder. It involved the capsule of Glisson as it entered the liver, and compressed its vessels. There was a mass through the posterior half of which ran, the abdominal aorta and vena cava.

The mass, then, was developed behind the mesentery, behind the stomach and pancreas, and in the soft tissues lying directly on the spinal column. The tumors were hard, and no one contained any softened material.

At the diaphragm, there was an interruption in these tumors going upward; but after leaving an inch or two of space unaffected, they began in the posterior mediastinum,—at first small, and reaching a large size, and passing forwards into the anterior mediastinum. The tumors on the left side were numerous enough, but small; while those on the right side were numerous and large, pushing the pleural fold of the mediastinum over the whole apex of the right lung;—one of these latter was as large as a good-sized peach. The pericardium presented a small group of these tumors on its right side.

The lungs were free from tubercles. The left appeared to be healthy, but the right was of dark color, considerably condensed, though not contracted, as if from long-continued mechanical congestion. The thoracic group of tumors weighed twelve ounces; the abdominal group, twenty-nine; many, however, were not removed.

The spleen was large, weighing fifteen ounces; was flattened and elongated downwards; being rather thick-set with rather hard, white tumors, some as large as a cherry. These were all imbedded in the organ, although some reached the surface. When cut into, these tumors were found to be compound, being composed of a group of several white masses, separated by septa of spleen tissue. The hilus was thick-set with small, white tumors, about the size of a buckshot. These were firmly attached to the organ, but not incorporated with it.

The colon contained a considerable quantity of a material like thick gruel, of a yellowish gray color, which appeared like pus nearly pure. Its mucous membrane was thick and fleshy, of the color of beef's muscle. The lining membrane of the stomach was not much thickened, but was red, and covered by adherent mucus. The thickened, muscle-colored mucous membrane continued up the whole course of the large intestine, and a short distance into the ilium. At the caput coli was a pretty large accumulation of this purulent fluid. The enlarged glands were numerous at the root of right lung posteriorly.

From the microscopical examination of the bodies thus far, Dr. C. was disposed to believe that they were tuberculous.

In conclusion, he thought that the exsanguinated condition of the patient, in connection with this disease of the spleen and lymphatics, seemed to prove that they were true hæmigenetic organs. Dr. Sayre, in relation to Dr. Clark's first specimen, was reminded of a similar case that occurred five years previous, where so large a mass of crude tubercle was expectorated that it lodged in the glottis, and tracheotomy had to be performed. Dr. Gonley stated that he saw such bodies in the tubercular cavities of a child's lung 17 months old. He had seen similar masses in the lungs of a snake.

### PERICARDITIS AND FATTY DEGENERATION OF KIDNEYS.

Dr. Cock presented the following, for a candidate :—

Mary Smith, æt. 33, a native of Germany, was admitted to the New York Hospital Jan. 3d, 1859,—during the service of Dr. Cock. The patient is of a fair complexion and phlegmatic temperament ; the mother of two children ; had enjoyed pretty good health until about two years ago ; when, after exposure to cold, she had a chill, followed by severe pain in the lumbar region, and a diminution in the quantity of urine. From that she recovered ; and, saving a puffiness of the face and occasional swelling of the feet, nothing unusual took place till about six months ago ; when she had an attack of hæmaturia. The treatment she received is unknown. It is said that she never had rheumatism, and there is no hereditary tendency to disease in the family.

The patient is somewhat emaciated ; cachectic and anemic, with a complexion rather of a waxy hue ; face puffy , and a strong mercurial fator of the breath, with a copious secretion of saliva ; surface dry ; œdema of lower extremities ; considerable dyspnœa ; pulse 75 ; and feeble headache, drowsiness, and dimness of vision ; has a lancinating pain in the precordial region, accompanied with palpitation of the heart ; tongue coated white ; appetite poor ; bowels regular ; decubitus dorsal. The urine is highly albuminous, sp. grav. 1011 ; but on microscopic examination, it was found to contain no fibrinous casts. Over the precordial region, a strong, purring tremor exists. By auscultation, a very marked double-friction sound, of varying intensity in different parts,—most noticeable near the base,—creaking, grazing, and musical (?) tones. Exocardial sounds too intense to recognize endocardial murmurs.

*Diagnosis.*—Hypertrophy; pericarditis with presumed endocarditis. Kidneys probably exhibiting morbus Brightii.

*Treatment.*—Emp. vesicat. 3+5 to precordial region, and opium q. s. to quiet at night.

January 5th. General condition a little improved; face less puffy; ptyalism about the same; only a *single* friction sound audible at the base of the heart; pulse irregular.

January 7th. Had an epileptiform convulsion this morning about 6 A. M.; and twitching of the muscles of the upper extremities continued for some hours after, with great restlessness and mental agitation. There is still a friction-sound heard at the base of the heart, and a systolic endocardial murmur at the apex. Ord., asafœtida, per rectum; and, ℞ Hoffman's anodyne ℥ ss., tinct. hyosciam. ʒ ij., am. tinct. val. ℥ ss., aqua camphoræ, ℥ iij., M.; of which she was directed to take a tablespoonful every two hours.

January 8th. The patient remained pretty quiet until night; when convulsions again set in; and after having three, she remained in a semi-comatose condition, with stertorous and sighing respiration. Passed 12 oz. of urine, per catheter, in twenty-four hours.

The asafœtida enema repeated; and ordered, sinapism to lumbar region, with emp. vesicat. to nape of neck.

Jan. 9th. Had another convulsion this morning, is still in a semi-comatose condition; eyes turned upwards and to the left; respiration stertorous and sighing; and she remained in this condition until about 2 o'clock A. M. on the morning of the 10th, when death took place.

*Post Mortem.*—10 hours after death:—

*Thorax.*—Lungs œdematous; the left not crepitant, and on section very little air could be pressed out; slight old pleuritic adhesions existed at the apex of the right. Blood flowed into the pleural cavities before we looked for any inflammatory effusion.

*Heart.*—Pericardium distended with clear serum; and a recent deposit of lymph existed on both its surfaces to a considerable extent, especially around the base of the heart; the aortic valves were opaque and slightly thickened; the mitral was also thickened and a firm or hard deposit of fibrine existed in the free extremity of its posterior fold; the muscular substance of the left ventricle was considerably hypertrophied; the weight of the heart, including the pericardium, being 20 oz.

*Abdomen.*—The *liver* was fragile and congested, but not enlarged, weight 3¾ lbs.

*Kidneys.*—Both together weighed only 7½ oz.; the granular portion being almost entirely converted into fat. All the other organs examined were in a healthy condition.

### Regular Meeting, Feb. 9, 1859.

### CANCER OF BREAST.

Dr. T. C. Finnell presented a specimen of cancer of the breast, removed a few days ago from a woman aged 35, the mother of two children. About two years ago, while nursing her first child, she noticed, for the first time, a small tumor on the lower portion of the left breast. The physician who saw it at that time advised her to leave it alone. Since that time it has grown gradually, the size increasing a great deal while nursing. Dr. F. saw her for the first time about six months ago, and recognized a scirrhus tumor. She was at that time nursing her second child, and she remarked that it was increasing quite rapidly. She complained also of a good deal of pain. Dr. F. advised the removal of the tumor; and it was accordingly done, on Sunday morning last. The whole of the gland was removed. The mother had nursed the child from the diseased breast up to a day or two before its removal.

There was a cyst nearly the size of a hen's egg on the surface of the mass, about its middle, which contained a dark-colored fluid. A cyst had ruptured a short time previous to the operation, which contained the same kind of fluid. Several enlarged lymphatics were removed from the axilla.

Dr. Clark asked how frequently cancerous degeneration took place in the breast of a woman while nursing. He was not familiar with its occurrence under such circumstances.

Dr. Wood stated that he had seen it grow quite rapidly just before childbirth. The increased quantity of blood which was sent to the gland at that time, offered a sufficient explanation.

Dr. Finnell next presented a specimen of a *femur*, which was the seat of *exostosis*. It was removed from a male dissecting-room subject, aged 40. The growth proceeded from the anterior surface of the bone, about two inches below the trochanter, pyramidal in form, the apex projecting downward and forward, through the muscular tissues in the neighborhood,

It was about 2½ inches in length, and seemed to have grown from above downwards into the substance of the muscles; so that when the subject lay upon the table, no deformity could be recognized. Nothing was known of the history of the case.

Dr. Jas. R. Wood presented a specimen taken from a patient who had been an inmate of the Bellevue Hospital, since April last. She was about 30 years of age, and entered the Hospital with chronic disease of the knee-joint. She has suffered about a year from synovitis; and when examined, after her admission into the Hospital, it was discovered that she had tubercles; and shortly before her death it was also discovered that she had Bright's disease.

She was treated for synovitis, in the usual way, by counter-irritation, drains in the neighborhood of the joint, and position on the double-inclined plane.

About a month before she died, it was discovered that there was displacement at the knee-joint; that the left condyle had lost its position, and occupied the position of the right;—in other words, subluxation of the joint had taken place, outwards and backwards. The outside of the condyle, it will be seen, said he, is bare of the periosteum, and the bare bone has, by attrition, so diseased the ligament that it had given way. This seems to be the only way that the luxation could be accounted for. We know that occasionally there is subluxation from injury, as described by Mr. Cooper; where the ligament gives way, and the patient falls at once, and is unable to get in position afterwards. He refers to a case where a lady, in attempting to get into a coach, turned her leg; the ligament gave way, she fell immediately to the ground, and was unable to raise herself in the erect position.

This specimen belongs to an interesting case; inasmuch as we find, from the ulcerative process, that the ligament is destroyed, and luxation is produced. It is rare to see luxation of the knee-joint from any cause, especially from ulcerative disease of the bone, affecting, secondarily, the ligament. We know in the hip-joint it is comparatively rare. Prof. Marsh, of Albany, who made a very thorough investigation of this subject, some time ago, and visited hundreds of museums both public and private, stated that he had never seen a case of luxation from ulcerative absorption of the hip-joint. We know now, however, that it does occur,—that he was mistaken; though the disease is very rare. There are some specimens in this museum, and others in private collections, that demonstrate this fact. He has two in his own museum. He brought this specimen because he thought it was a rare one, and was interesting to those gentlemen who do surgery.

Dr. Sayre stated that he had, within the last four years, in synovitis of the knee-joint, kept the limb in the straight position, and was perfectly satisfied of its utility. He asked if it was possible, in that position, that such a dislocation would have taken place. He thought that such a condition of things was materially facilitated by the ordinary, semiflexed position over the double-inclined plane.

Dr. Wood said that a good surgeon always imitated nature; and that the semiflexed position was the most easy and comfortable.

Dr. Sayre remarked that in the semiflexed position the tendons were apt to become contracted, and give rise to a good deal of pain. In these cases he thought it was better to divide them, and bring the limb out as nearly as possible in the straight position. He had seen gratifying results follow such treatment.

Dr. Wood had treated scores of cases on the double-inclined plane, and had never seen any bad result following. The subluxation was altogether an accidental circumstance, and had nothing at all to do with the double-inclined plane. He stated that the result coveted in these cases was anchylosis, and the limb was in the very best position for any such result, when over the double-inclined plane. He flexed the limb at the angle of 30°, and had so far found it to answer every purpose, both so far as progression and general comfort to the patient were concerned. Position, in these cases, was every thing. This was strikingly exemplified in the rectangular position of the elbow-joint. He stated that the leg flexed to the angle of 30°, was in a position that rendered the muscles passive. The same was the case with the position in anchylosis of elbow; and contraction of the muscles was not apt to take place.

Dr. Sayre observed that all the cases of anchylosis that he had seen, had been too angular. In regard to the position laid down in the books, he did not seem to agree with the authorities upon the subject. He made an exception in the case of the elbow.

Dr. Buck stated that he had known, in a case of luxation of knee from violence, that the lateral ligament, instead of rupturing, would tear out the surface of the condyle into which it was inserted. He said also that, in luxation of the foot outwards, with fracture of fibula, that the internal lateral ligament often, instead of being ruptured, tore off the extremity of the internal malleolus.

Dr. Dalton asked Dr. Wood if such a luxation would not likely be produced by muscular action.

Dr. Wood. No, Sir.

Dr. Buck stated that the displacement that he had noticed, as resulting from disease of the knee-joint, was dislocation outwards, by which the inner condyle of the femur was rendered very prominent; the leg, at the same time, being rotated outwards on its axis, and abducted on the thigh.

He observed that, uniformly, as the change in the relations of the knee-joint from white-swelling.

Dr. Sayre thought that Dr. Wood's dislocation was of the same character as those referred to by Dr. Buck.

### REGULAR MEETING, March 9, 1859.

#### OVARIAN CYST.

*Dr. Geo. A. Peters* presented a specimen of ovarian cyst weighing 21½ pounds, and a cancerous liver weighing eight pounds, removed from the body of a woman 50 years of age. He knew nothing of the history of the case, save that the disease first began to make its appearance last June. The disease of the ovary was confined to the right side, and was multilocular in its character. Its attachments were pretty general throughout the surface of the peritoneum. Six quarts of slightly turbid serum were found in the cavity of the peritoneum. Some of the cysts having been opened, were found to contain serum, while others were filled with a cheesy material.

The lobe of the liver was the seat of infiltrated cancerous degeneration, while the surface of the right lobe was covered with cup-shaped tumors. The cancer was of the scirrhous variety.

*Dr. Sims* next presented a specimen for a candidate,—

#### NECROSIS OF FIBULA.

*Exsection of the Fibula for Necrosis with Caries, which had existed fifteen months, in a boy aged ten years. Cured in three months, and the action of the limb entirely recovered.* By EDWARD B. TURNIPSEED, M. D., &c.

On February 3, 1858, I was called to see J——— G———, æt. ten years. The history of the case, as near as I could learn, was, that fifteen months previous to this date, while at play with some boys, he fell and hurt the left side of the left leg, and from then complained of pain, and was unable to walk without limping. The day following, the pain increased, accompanied with high fever. The family physician was sent for. It seems from what I could gather, that no diagnosis was given. The prescription, however, was flax-seed poultices, renewed several times during the day, and rest in the recumbent position. The patient grew no better; and after the expiration of a week or ten days, it was determined to call in a consulting surgeon. On palpation, matter was detected in the neighborhood of the point at which he first complained of pain, and where the poultices had been applied,—about the union of the superior with the middle third of the fibula. An incision was made, and pus flowed freely. The poultices were continued, and the patient put on constitutional treatment; the tincture of iodine was used.

The limb became more inflamed, extending downwards; abscesses forming at several points between the first one and the ankle joint. The bone after several months, was found to be very extensively diseased. During this time, some dozen physicians and surgeons had been consulted (amongst that number those who stand first in the ranks of surgery); all of whom advised the parents against an operation, and abandoned the patient to nature—*vis medicatrix naturæ*—and constitutional treatment.

*Examination.*—I found the leg about twice its normal size; four or five openings along the line of the fibula, from its head to the ankle-joint; caries of the bone its entire extent, save the external malleolus,—one inch and a half of its lower extremity. There was a small spicula of bone that had pierced the skin, and could be seen externally. This was near the head of the bone, and was found to be a large sequestrum which was so firmly retained in its position by its involucrum that it was impossible to produce the least motion. With the use of the silver probe and the point of the finger, I soon found the involucrum also extensively diseased.

I determined to remove the bone; but, as the boy, from the great drain upon his system, protracted for more than a year, had grown very pale, with colorless lips, and as his light hair, pigeon-breast, together with other evidences, indicated his scrofulous diathesis, I concluded to postpone it until he was in better condition to bear it. For this purpose, in addition to iodide of potassium, I prescribed nutritious diet, as well as wine.

On the 28th of February, 1858, the operation was performed. Drs. Louis Fassitt, Joseph Worsten, J. K. Smith, P. Bryce, and others, were present, and kindly rendered their assistance.

After putting the patient completely under the influence of chloroform, the operation was commenced by making an incision along the posterior border of the fibula, from opposite its articulating surface with the tibia, to within one inch and a half of the lower terminus of the external malleolus. I then prolonged my incision at its superior extremity, upwards and outwards, forming an obtuse angle, below and parallel with the anterior tibial nerve. Having laid bare the muscles, by cutting through the skin and cellular tissue the entire extent of the first incision, I commenced carefully dissecting the attachments of the extensor longus digitorum, biceps femoris, peroneus longus, soleus, and tibialis posticus, from the head of the bone; an aid, at the same time, drawing the anterior tibial nerve upwards and inwards, out of the way of the knife. This being completed, the anterior-superior and posterior-superior ligaments were severed from their attachments to the head of the fibula. A strong strip of tape was now passed between the bone and soft parts, and forced down, following the bistoury, as it severed the attachments of the extensor longus digitorum, peroneus longus, soleus, flexor longus pollicis, tibialis posticus, peroneus brevis, and the interosseus membrane, to the lower extremity of the first incision, one and a half inches from the inferior extremity of the external malleolus. Here, by means of the chain-saw, the bone was severed and the exsection completed.

During the entire operation there were no nerves injured, and only one of the small divisions of the peroneal artery to ligature. The edges of the wound—which was very extensive—were drawn together and kept in juxtaposition by means of adhesive strips and roller bandages; the limb placed on pillows somewhat elevated, and made permanent in one position.

The patient being aroused, remarked that he had felt no pain whatever, and knew nothing of what had been done. He now, however, suffered much pain from the length as well as depth of the wound. Prescribed gr. $\frac{1}{10}$ of sulphate of morphine every hour, until relieved.

Feb. 24th. Skin hot, tongue slightly coated, pulse 130, with no pain, but restless. Prescribe the continuation of the morphine, whenever suffering; also, ℞. hyd. cum creta gr. v. sulph. quin. gr. iij. M. fiat cht. 1, given at bedtime, and followed in the morning with seidlitz powders.

Feb. 25th. Patient quiet, having rested well with the opiate. Skin not so hot, pulse 132, tongue improved, with the desired effect of the medicines, and no pain. Sulph. morph. continued whenever suffering, and beef tea as a diet.

Feb. 26th. Slept very well, skin normal, tongue clean, and pulse 104, with good appetite. For the first time, since the operation, the wound was dressed. The bandages and compresses having been saturated with venous blood, which, being dry, made them difficult to remove. The wound, on removal of the adhesive strips, exhibited union by first intention its entire extent, with the exception of about half an inch at its lower extremity, which was kept open by a tent, to let escape any matter that might form. At this point there was a slight sero-purulent discharge. After cleansing the parts, and using ceratum simplex over the incised edges, the dressing was the same. Prescribed—℞ *externa*, liquor sodæ chlorinatæ ℥ ss., aqua distil. ℥ iv. M. used as a disinfectant, over the bandages. Diet: Stewed or roasted oysters, beef tea, and a half wine-glassful of port wine three or four times a day.

Feb. 27th. Slept well, no pain in the operated limb, tongue clean, skin normal, and pulse 104. The bandages being removed, I found the parts the same, except less sero-purulent discharge. After sponging the parts, a solution of chloride of soda was injected around the end of the remaining portion of the exsected bone. Dressing, idem; diet, idem.

Feb. 28th. Not doing so well. Prescribed—℞. Hyd. sub. mur.; pulv. Doveri,—aa. gr. iij. M. fiat cht. 1. Given at bedtime.

March 1 and 2. Patient better, pulse 100, with all the symptoms improved. Very little discharge of matter from the wound, with a more healthy color. A solution of nitrate of silver ʒ i. to ℥ iv. of water, was used as a means of producing healthy granulation. The iodide of potassium was renewed. Diet: beef steak, mutton chops, and the quantity of wine increased.

He was visited regularly, and found to be improving at each visit; and it was not known until the 6th of March, that there would have to be some little exfoliation from the inner edge of the portion of the bone yet remaining. This exfoliation continued until about the 15th of May, when it was considered ended. The patient (although a small point was kept open, to prevent any burrowing of matter) was permitted to walk about the house; at first, assisted; and afterwards, alone. There being no more exfoliation, the opening was closed, about the 1st of June; the patient pronounced cured, and dismissed.

It has now been more than a year since the operation was performed, and there has been neither a return nor any symptoms of a return of the disease; and the boy has never enjoyed better health,—running, leaping, jumping, and walking, as well as he ever did. So perfect, indeed, are the results of the operation, that it is considered, by all who have either seen the boy, or heard of his perfect use of the limb, as unique.

*Pathological appearances of the removed bone.*—Its length is seven and one-fourth inches. Its circumference, at its lower extremity, is two and a half inches; at its center, three and three-fourths inches; and at its superior extremity, two and a half inches. The length of the sequestrum is five inches; and its circumference, one and one-fourth inches. The superior third of the involucrum is attached to the middle third by ligamentous union. The whole length of the posterior portion of this bone—the involucrum—is denuded of its periostum. A probe can be passed from one end to the other, through the center, its entire extent, which is a mass of disease.

*The Difficulties as well as Dangers of the Operation.*—M. Velpeau,*

---

* Velpeau, *Anatomie Chirurgicale*, page 615.

Professor in the College at Paris, first pronounced it dangerous to amputate above the tubercle of the tibia, for fear of opening the articulation of the knee-joint; and afterwards, M. Zenoir* proved, by a number of dissections, that the synovial cavity of the knee-joint is continuous with the superior tibio-fibular articulation, once in every four persons; and that once in every two persons this membrane descends to the head, or behind the head, of the fibula; and that then there is a communication between the knee-joint and the bursa mucosa, situated between the ligamentum patellæ, near its insertion, and the front of the tibia. As we know, the synovial membrane of the knee-joint is by far the most extensive in the skeleton; and, therefore, the dangers under the above circumstances, of exposing so large a surface to inflammation.

This operation was first proposed by Desault; and, afterwards, a portion extracted by Seutir, for necrosis. He, however, found it unnecessary to disarticulate the head of the bone. Also, the external malleolus was left. He found it necessary to ligature a number of arteries, and to cut the external popliteal nerve. His patient was well at the expiration of four months, and the use of the limb almost recovered.

M. Malgaigne,† Professor of Operative Surgery in the College at Paris, had occasion to extract portions of this bone twice. First, the superior third; and afterwards, the head of the bone; the former for caries, and the latter for cancerous degeneration. He says, "We necessarily cut the anterior tibial nerve, which turns around the head of the fibula;" and that, in both of his cases, "there was paralysis, and turning downwards and inwards of the point of the foot."

*Deductions.*—In diseases of the bones, where the patients are of a scrofulous diathesis, they should not, because of this misfortune, be left or abandoned to nature—*vis medicatrix naturæ*—for a cure, to linger and suffer, and to become burdensome, as well as loathsome, to their families and friends, for years and perhaps life. And that the fibula can be exsected or resected, without the bloody operation of Seutir, and without the paralysis of Prof. Malgaigne; that we do not necessarily have to cut the anterior tibial nerve; but that it may be always avoided, and the recovery and use of the limb as perfect as it ever was.

---

* *These de concours pour l'agrégat*, 1835.

† Malgaigne, *Médicine Operatoire*, page 238.

### NECROSED BONE.

Dr. Jas. R. Wood presented a specimen of *necrosed bone*, which was taken from a man, who, four months before, suffered amputation of the thigh, for compound fracture of the leg. The wound healed soon after. The amputation was performed below the junction of the middle and lower thirds of the thigh. Shortly after it had closed over, dead bone began to show itself; and it was found that the whole of the circumference of the lower portion of the femur, was in a necrosed condition; which gradually extended, the separation of the periosteum being encouraged by the frequent introduction of probes, &c., between it and the bone. Very soon, new bone began to be deposited from this periosteum, which has increased until the present, when about four and a half inches of the distal extremity of the bone was removed with a pair of duck-bill forceps. The new deposit is enormous, being about four times the natural size of the bone.

In connection with this specimen, he stated that Dr. Markoe looked upon the cause of necrosis in that locality, as owing to a want of nutrition of the bone, the supply of blood from the medullary artery having been cut off; and referred to a very clever paper, by that gentleman (Dr. M.), which was published in a recent number of the *New York Journal of Medicine.*

### NECROSIS OF TIBIA.

The next case was a leg and portion of the thigh, which he removed three weeks ago, from a boy eight years of age. In September last, he was kicked on the shin by one of his playmates; inflammation followed, and the family physician applied leeches, but with little benefit. Poultices were afterwards kept applied; but the little patient suffered so much from pain in the part, that Prof. Parker was called, at the end of a fortnight. He made an incision two or three inches in length, and found dead bone to exist. Dr. Parker saw the patient only once; and the physician continued his attendance until Dr. Wood was called to the case. On examining the limb, he found that there was necrosis of the tibia, trom the tubercle to within half an inch of the internal malleolus. He commenced enucleating the bone from the periosteum, and watched with interest the favorable progress of the case, until the patient was seized with severe pain in the joint, terminating with suppurative inflammation, rendering amputation imperative. Previous to the operation, however, the joint was opened, a large quantity of pus being discharged.

The specimen showed that the whole of the anterior and lateral portion of the tibia was necrosed, and that new bone had formed around the sequestrum. Had it not been for the unfortunate complication at the lower end of the bone, all of the dead bone would have been removed, and new bone would have occupied its place. A longitudinal section of the bone, showed a cavity to exist, both at its upper and lower end, which was filled with tuberculous matter, and a cancellated sequestrum, as was proved by microscopical examination. He stated that the specimen proved that the deposit of new bony matter was greater in extent and amount, than the dead material. In this particular, it was analogous to many that he had reported. The wound, after the amputation, healed rapidly, and was closed up at the end of twenty days.

Dr. Markoe, in reference to the explanation of the formation of sequestrum, stated that he had examined over fifty femora, in reference to the point at which the nutritious artery entered the bone. In about half the cases it entered at or below the middle of the femur.

In a certain number of cases the nutritrious arteries are double; and in all cases they enter from below, obliquely towards the upper extremity. He stated also, that the artery, previous to entering the bone, traverses a greater or less extent of muscular tissue, as the case may be; and hence, it was not impossible for the artery to branch from the femoral, at least as low down as the middle third. Sequestra similar to the one presented by Dr. Wood, he stated, were most common in the femur, radius, and ulna; the nutritious arteries in these cases, the direction being from below upwards, were liable to be cut or sawn in amputation; hence, the formation of such sequestra at the end of the bone. In the tibia, fibula, and humerus, the nutritious artery enters from above downwards, and consequently is not implicated in amputations. He had never seen a sequestrum from the humerus.

Dr. Dalton.—Is ligature of the femoral liable to be followed by necrosis?

Dr. Markoe answered in the negative. He stated further, that the second specimen was a very interesting one. It showed that the bone was dead for two-thirds of its extent; which is generally the case when disease is the result of an injury. The disease, at the lower end, had extended through the cartilage of incrustation. This state of things occurs now and then in the knee-joint, but most frequently at the tibio-tarsal articulation. An amputation under such circumstances was imperative.

Dr. Wood presented a third specimen, taken from a man sixty years of age, who entered Bellevue Hospital on the 17th of last September, with compound fracture of the tibia and fibula, the former bone being exposed to the extent of four inches. At the expiration of a fortnight, this portion of the tibia was removed without any periosteum being attached to it. The patient went on doing very well, the wound healing nicely, until the following November, when the parts became involved in a low grade of inflammation, in consequence of which, the limb was amputated. On examination of the limb, after removal, a gratifying condition of things presented itself. Notwithstanding four inches of bone had been removed, comprising the whole circumference of the tibia, the space was occupied by new bone, formed from the periosteum, which was left after the enucleation. The callus was tolerably firm, and would have, in due time, united the upper and lower fragments. He stated that, had the periosteum surrounding the detached fragment been removed, there would have been no deposit; but by leaving it, this restorative process threw out new bony material; and the patient would have had a very good and useful leg, had not the unhealthy suppuration taken place. He further stated that, had this process been continued, the new matter deposited would have been greater in quantity and extent than the original piece of bone removed. He said that, in his judgment, the time would come, when by careful and patient treatment of these cases, very few legs would be amputated; at least surgeons would be induced to save a good many more than they now do. He recollected when he was a student, that amputation was a great deal more common than at present. Every case of compound comminuted fracture of the ankle was amputated; in fact, many limbs were condemned at the very outsets, any attempt at restoration being considered out of the question; and that was the practice up to a very few years ago. He stated that the proper way in these cases was to coax nature to do her part; the success of such an endeavor was strikingly exemplified, in the treatment of the fractures in the New York Hospital; especially by Dr. Buck's bran-splint. It is fortunate for the patient, and creditable to the physician, that conservative surgery is in the fashion.

Dr. Finnell next presented a deformed sternum and costal cartilages, removed from a woman who died of phthisis. The specimen was interesting, on account of the deformity which it presented. The bone was crowded backward, being only two inches distant from the spinal column, the costal cartilages as a consequence, arching backwards. The cause he assigned to the prevailing custom of tight lacing.

